# Collagen I but not Matrigel matrices provide an MMP-dependent barrier to ovarian cancer cell penetration

**DOI:** 10.1186/1471-2407-8-223

**Published:** 2008-08-05

**Authors:** Katharine L Sodek, Theodore J Brown, Maurice J Ringuette

**Affiliations:** 1 Department of Cell and Systems Biology, University of Toronto, Toronto, Canada; 2 Department of Obstetrics and Gynecology, Samuel Lunenfeld Research Institute, Toronto, Canada

## Abstract

**Background:**

The invasive potential of cancer cells is usually assessed *in vitro *using Matrigel as a surrogate basement membrane. Yet cancer cell interaction with collagen I matrices is critical, particularly for the peritoneal metastatic route undertaken by several cancer types including ovarian. Matrix metalloprotease (MMP) activity is important to enable cells to overcome the barrier constraints imposed by basement membranes and stromal matrices *in vivo*. Our objective was to compare matrices reconstituted from collagen I and Matrigel as representative barriers for ovarian cancer cell invasion.

**Methods:**

The requirement of MMP activity for ovarian cancer cell penetration of Matrigel and collagen matrices was assessed in 2D transwell and 3D spheroid culture systems.

**Results:**

The broad range MMP inhibitor GM6001 completely prevented cell perforation of polymerised collagen I-coated transwell membranes. In contrast, GM6001 decreased ES-2 cell penetration of Matrigel by only ~30% and had no effect on HEY cell Matrigel penetration. In 3D culture, ovarian cancer cells grown as spheroids also migrated into surrounding Matrigel matrices despite MMP blockade. In contrast, MMP activity was required for invasion into 3D matrices of collagen I reconstituted from acid-soluble rat-tail collagen I, but not from pepsin-extracted collagen I (Vitrogen/Purecol), which lacks telopeptide regions.

**Conclusion:**

Matrigel does not form representative barriers to ovarian cancer cells in either 2D or 3D culture systems. Our findings support the use of collagen I rather than Matrigel as a matrix barrier for invasion studies to better approximate critical interactions and events associated with peritoneal metastasis.

## Background

Cancer cell invasion of tissue matrices is a fundamental aspect of metastasis. Extracellular matrices (ECM) are generally considered to be of two types, basement membrane and stromal/interstitial. Basement membrane matrices are normally deposited beneath epithelia, and its components characteristically include collagen IV, laminin, perlecan and nidogen, which interact to form a thin, dense, cross-linked polymeric network with high tensile strength. Stromal/interstitial matrices form the majority of the body connective tissue and are composed primarily of fibrillar collagen I that is cross-linked into a stable meshwork to impart 3D structural support. As both basement membrane and stromal matrices present a steric barrier to cell transmigration, matrix remodelling is a necessary and critical contributor to metastasis. Tumour cells acquire the ability to surmount ECM barriers by expressing a range of proteases [1], particularly members of the matrix metalloprotease (MMP) family [2-4]. MMPs are vital for the degradation of both basement membrane and stromal matrices: the gelatinases MMP-2 and MMP-9, and transmembrane MMPs are critical mediators of basement membrane remodelling [5,6], whereas the cleavage of stromal fibrillar collagen I networks is limited to MMPs-1, -8, -13 and the transmembrane MMPs [2].

*In vitro *assays are valuable for evaluating the potential role of candidate modulators of invasive behaviour, particularly in the present era of high throughput proteomic and genomic screens which are identifying large numbers of possible therapeutic targets. Cancer cell invasion is typically assessed *in vitro *using the transwell Matrigel invasion assay. Matrigel, an extract derived from mice harbouring Engelbreth-Holm Swarm (EHS) tumours, is rich in laminin and collagen IV and is therefore used as a surrogate basement membrane for investigating a variety of cell behaviours, including cancer cell invasion [5,7]. For invasion assays, a thin layer of Matrigel is coated onto a porous membrane in Boyden or Transwell chambers and cell penetration is assessed. As such, the assay is considered to be a reliable and valuable test to evaluate cancer cell invasiveness [5,8-11]. In an assay similar to the Matrigel chemoinvasion assay, transwell membranes can be coated with collagen I to reflect cellular invasion through the confines of stromal/interstitial matrices.

For cancers such as ovarian, gastric and colon, which metastasise within the peritoneal cavity, it is paramount that the *in vitro *models adequately reflect the processes that occur during peritoneal dissemination. Epithelial ovarian cancers (EOC) are the most deadly of the gynaecological cancers and are the fifth leading cause of cancer-related deaths in North American women [12]. The majority of EOCs metastasize locally in a manner that does not involve haematological transport. Ovarian tumour cells exfoliate and are carried via peritoneal fluid to secondary sites in the abdominal cavity where their attachment, invasion of the submesothelial connective tissue, and proliferation form peritoneal deposits. An inflammatory response typically accompanies disease progression and alters the peritoneal membrane in a manner that renders it prone to cancer cell adhesion [13,14]. This further facilitates tumour dissemination such that a self-promoting vicious cycle of metastasis ensues and inevitably leads to impaired functioning of abdominal organs: the obstruction and malfunctioning of the gastrointestinal tract are a frequent cause of morbidity from ovarian cancer [15,16]. Devising effective strategies to prevent further metastatic spread is instrumental for improving survival of patients diagnosed with advanced disease. Important cell behaviours that contribute to ovarian cancer disease progression include adhesion (cell-cell and cell-matrix), migration, and invasion [17].

The surfaces of the peritoneal cavity are covered by a layer of mesothelial cells that function in an antiadhesive manner to promote gliding of the abdominal viscera [18]. The mesothelial layer also protects against cancer cell attachment [19], as it conceals the underlying connective tissue matrix to which tumour cells preferentially attach [19,20]. Collagen I is present in abundance beneath the peritoneal mesothelium [21]. The collagen-binding integrins α2β1 and α3β1 mediate *in vivo *peritoneal metastasis of gastric tumour cells [22,23], and their importance in this process has been inferred for ovarian cancer cells [24-27]. Furthermore, collagen I is the preferred substrate for ovarian cancer cell attachment [28], and stimulation of motile [25,29] and invasive [24] behaviour.

In these studies we compared the performance of Matrigel and collagen I substrata as *in vitro *invasion matrices, both in 2D (planar) and 3D contexts. In particular, we sought to determine whether cell penetration of these matrices required MMP activity, reflecting the mechanisms needed for cancer cell invasion *in vivo*. We show that in contrast to the invasion of collagen I matrices, MMP-mediated proteolysis is not required for cell penetration of Matrigel for ovarian cancer cells in either 2D transwell or 3D spheroid cell invasion systems. This contrasts with the requirement of MMP activity for the invasion of basement membranes *in vivo*, indicating the limitations of Matrigel for the evaluation of cancer cell invasion.

## Methods

### Cell culture

Four human ovarian cancer cell lines were used in these studies: HEY and HOC-7 cells (obtained from Dr. A. Marks), OVCA429 cells (from Dr. R. Kerbel), and ES-2 cells from American Type Culture Collection (Manassas, VA). Cells were maintained in α-minimal essential media (α-MEM; GIBCO) supplemented with 10% fetal bovine serum (FBS; Cansera International Inc), 0.017% penicillin G and 0.01% gentamycin in a humidified incubator at 37°C and 5% CO_2_.

### Transwell matrix penetration assays

Transwells of 8 μm pore size (Costar, Corning Inc., Corning, NY) were coated with matrix, or left uncoated, for simple migration assays. Matrigel (VWR CanLab, Missisauga, ON) was diluted in ice-cold PBS to 175 μg/ml and 200 μl were added to transwells for a total of 35 μg per well. The experiments spanned three lots of Matrigel and included both growth factor-reduced and phenol red-free preparations. Results obtained were consistent with all three lots Vitrogen (Cohesion, Palo Alto, CA, now sold as Purecol) was neutralized with NaOH, diluted to 200 μg/ml and 100 μl were added to each transwell. Matrix solutions within transwells were polymerised at 37°C for 1 hr then dried onto the transwells overnight at room temperature. For examination of matrix integrity, a subset of coated transwells were biotinylated under sterile conditions, using 20 μg/ml EZ-Link Sulfo-NHS-LC-LC-Biotin (Pierce, Rockford, IL) in 50 mM sodium bicarbonate, pH 8.3 for 2 hrs, and subsequently quenched using 50 mM Tris-HCl, pH 7.5. Biotinylated matrices were washed 3 times prior to addition of cells. Matrices were equilibrated in serum-free medium prior to addition of 2 × 10^5 ^cells in medium containing 1% FBS. Outer wells initially contained serum-free medium that was replaced by 10% serum-containing medium (chemoattractant) after an initial 1–2 hrs that allowed for cell attachment. The broad-range MMP inhibitor GM6001 (Chemicon International Inc.) (25 μM) or the DMSO carrier (as control) were applied to both upper and outer wells at the time of cell seeding. Additional cells were seeded into tissue culture dishes to verify that GM6001 treatment did not affect cell proliferation within the duration of the assays. Following a 24–72 hr incubation to allow cell penetration, total invaded cells were quantified based on nucleic acid measurement. Matrix integrity was assessed by confocal microscopy.

#### Quantification of invaded cells

A simple unbiased method was used to quantify total cells that had invaded through each transwell. Excess media was aspirated from the transwells, and following a brief rinse in PBS, tranwells were placed in fresh wells containing 500 μl trypsin (0.02–0.1%) so that the invaded cells on the underside of the transwell were released into this solution within 5 min. These solutions were microcentrifuged (2000 g, 5 min), and pellets containing the invaded cells were frozen (-70°C). Quantification of invaded cells was performed based on nucleic acid content using CyQUANT™ dye (Molecular Probes, Inc., Eugene, OR) according to manufacturer's instructions. For comparison, a subset of the transwell membranes were fixed in 4% paraformaldehyde, rinsed in PBS, and stained with DAPI (10 min). Non-invaded cells were swabbed from the upper side of the membrane. The membrane was mounted and invaded cells were visualized using fluorescence microscopy at low magnification.

As a note, we initially used the traditional DAPI staining microscopy-based technique to quantify invaded cells, but observed high regional variability (non-uniform cell penetration) regardless of whether Matrigel or collagen I coating was used, with the majority of transmigrated cells found in the central region. This phenomenon appears inherent to the coating process, as it also occurs when commercially coated transwells are used (BD biocoat product literature) and likely results from a meniscus effect causing higher deposition of matrix near the edges of the transwell. The CYQUANT quantification described above circumvented the large variability as well at the potential for selection bias that is inherent to the traditional DAPI counting procedure.

#### Confocal Microscopy

For examination of matrix integrity, invasion assays were performed using transwells coated with biotinylated matrices. These were fixed for 30 min in 4% paraformaldehyde, stained with FITC-streptavidin (Molecular Probes Inc.) diluted 1:300 in 2% BSA/PBS, mounted in anti-fade medium (1% DABCO, [Sigma-Aldrich], 90% glycerol, 10% PBS) and visualized by confocal laser scanning microscopy (LSM 510, Carl Zeiss Inc., Toronto, ON). In addition to 16× and 40×, matrices were examined under low power (10×), so larger portions of the transwell (25%) could be visualized to ensure images recorded under high power were truly representative.

Generally, three experiments were performed for the transwell assays, with three replicates each, for each cell line. Results were highly consistent between replicates within each experiment, and between experiments thus the data were pooled between the individual experiments and subjected to statistical analysis to yield the results shown.

### Scratch wound migration assay

Cell migration in the absence of a requirement for cell-cell detachment was assessed using a scratch wound assay. Monolayers of confluent cells in a 6-well plate were wounded by scraping with a P1000 plastic pipette tip and rinsed twice with PBS to remove floating cells. The underside of the dish was marked to indicate the wounded area where the initial photos were taken. Subsequent images were periodically recorded at the same location over the next 18 hrs with an inverted phase microscope/PixelLink megapixel FireWire camera (Vitana Corporation, Ottawa, ON). Cell motility was evaluated by the reduction in distance between opposing edges of the wound.

### 3D spheroid invasion assays

Spheroid cell culture was performed using the hanging drop method [30]. Briefly, 20 μl droplets of culture medium containing 5 × 10^4 ^cells were suspended from the lid of tissue culture dishes for 72 hours, during which time cells clustered into compact sphere-like formations. The spheroids were then embedded in gels. Matrigel was used at either full concentration (~11 mg/ml) or diluted in cell culture medium to a final concentration of 4 mg/ml. Two collagen preparations were tested: commercially available bovine pepsin-digested Vitrogen, and acid-extracted rat tail collagen I (a gift from Dr. J. Sodek), both stock solutions were 3 mg/ml in 0.012 N HCl. Collagen I solutions were neutralized on ice with 0.1 N NaOH and diluted to a final concentration of 2.1 mg/ml containing 5% FBS using [10×] α-MEM. Matrix solutions were coated onto the bottom of 96-well plates (75 μl) and polymerised to form a base upon which spheroids were pipetted (1–2 per well). The extra medium transferred was removed using sterile blotting paper, and then an additional 100 μl of matrix was applied to encase the spheroids. Images were recorded initially and at 12–24 hr intervals thereafter using an inverted phase microscope. Spheroid invasion was qualitatively assessed as either positive or negative. Sequential images were compared/inspected for invading cells, which appeared as a corona around the original spheroid that expanded with time.

## Results

### MMP activity is necessary for cell penetration of collagen I-but not Matrigel-coated transwells

**Figure 1:**
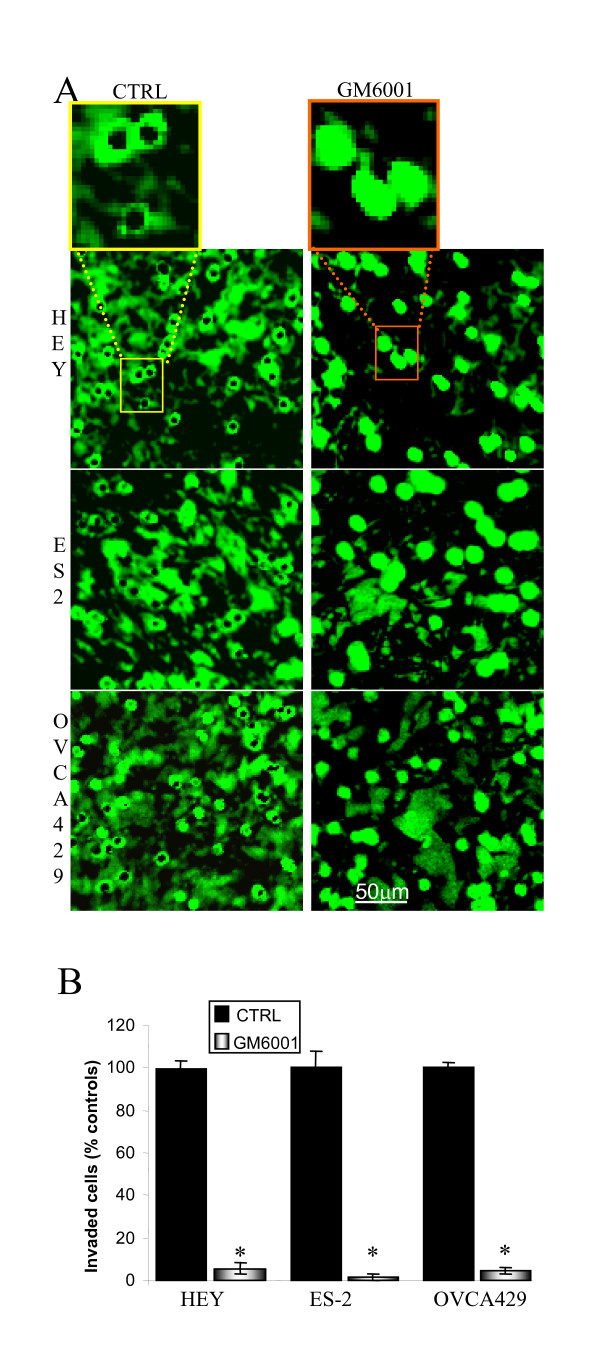
**Transwell collagen I invasion is MMP dependent.***A*, Confocal microscopy of biotinylated collagen I-coated transwells shows HEY, ES-2 and OVCA429 cells clearance of collagen I from transwell pores is abrogated by the MMP inhibitor GM6001. Transwell membranes coated with collagen I (0.6 μg/mm^2^) were biotinylated prior to seeding of cells. Membranes were and fixed and stained with FITC-streptavidin at assay termination. Representative areas recorded at 16× magnification are shownwith a magnified view of the transwell pores shown above. *B*, Collagen I transwell invasion was abbrogated by MMP inhibition. Invasion assays were terminated at 24 hrs for HEY and 55 hrs for OVCA429 and ES-2. Bars represent the mean ± S.E. *Significantly different from control (p < 0.05; 2-tailed t-test).

Coating transwell membranes with 20 μg of polymerised collagen I (Vitrogen) resulted in the formation of a barrier that required MMP proteolytic activity for cancer cell penetration (Fig. 1). Confocal microscopy of the coated transwell membranes revealed that the polymerised collagen I matrix solution had pooled within the transwell pores, forming plugs. The ability of HEY, ES-2 and OVCA429 cells to clear the collagen I within pores was prevented by the broad range MMP inhibitor GM6001 (Fig 1A). Consistent with the MMP dependence for collagen I clearance, cell invasion through the matrices was abrogated (>95%, p < 0.05; with actual p-values of 1.6E-6, 1.9E-5 and 1.2E-4 for HEY, ES-2 and OVCA429 cells respectively) in the presence of GM6001 (Fig 1B). Although GM6001 usually caused a complete abolition of cell invasion through collagen I matrices, a few cells occasionally traversed the membrane, which we speculate occurred as a result of rare pores being incompletely blocked due to irregularities in the transwell membrane itself (double holes) or the presence of air bubbles formed during matrix polymerisation.

**Figure 2:**
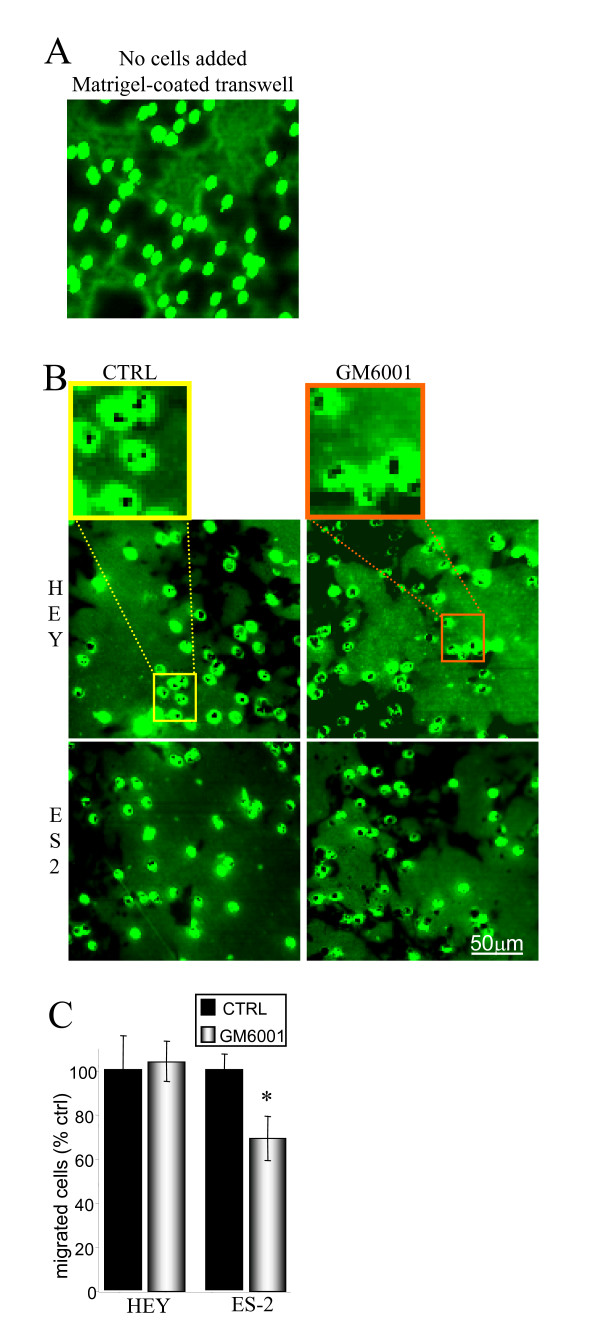
**Matrigel penetration does not require MMP-mediated proteolysis.** Transwell membranes coated with Matrigel (1.0 μg/mm^2^) were biotinylated prior to seeding of cells. Membranes were and fixed and stained with FITC-streptavidin at assay termination. Representative areas recorded at 16× magnification are shown. *A*, Control membrane (no cells applied) reveals the Matrigel pools within and occludes the transwell pores, in a similar manner to the collagen I matrix. *B*, Confocal microscopy of biotinylated Matrigel-coated transwells indicates HEY and ES-2 cell perforation of Matrigel-plugged transwell pores is unaffected by MMP blockade (GM6001). A magnified view of the transwell pores is provided above. *C*, Transwell Matrigel penetration was not prevented by MMP inhibition. Cell quantification was performed 55 hrs after seeding. Bars represent the mean ± S.E. *Significantly different from control (p < 0.05; 2-tailed t-test).

When transwell membranes were coated with polymerised Matrigel, the matrix concentrated within pores, forming plugs in a manner similar to the collagen I (Fig. 2A). However, in contrast to polymerized collagen I, cell perforation of the Matrigel plugs was not prevented by MMP inhibition (Fig. 2B). The extent of HEY cell penetration was not lowered by MMP blockade (p = 0.87; Fig. 2C) indicating that these cells are able to transverse Matrigel matrices in absence of MMP activity. GM6001 reduced ES-2 cell penetration by approximately 30% (p = 0.01); a modest effect as compared to the complete abrogation of cell penetration through collagen I. Our preliminary studies with OVCA429 cells indicated a results similar to ES-2 with only modest reductions in Matrigel penetration in response to GM6001 (data not shown).

### MMP blockade can inhibit transwell migration through mechanisms unrelated to Matrigel coating

**Figure 3:**
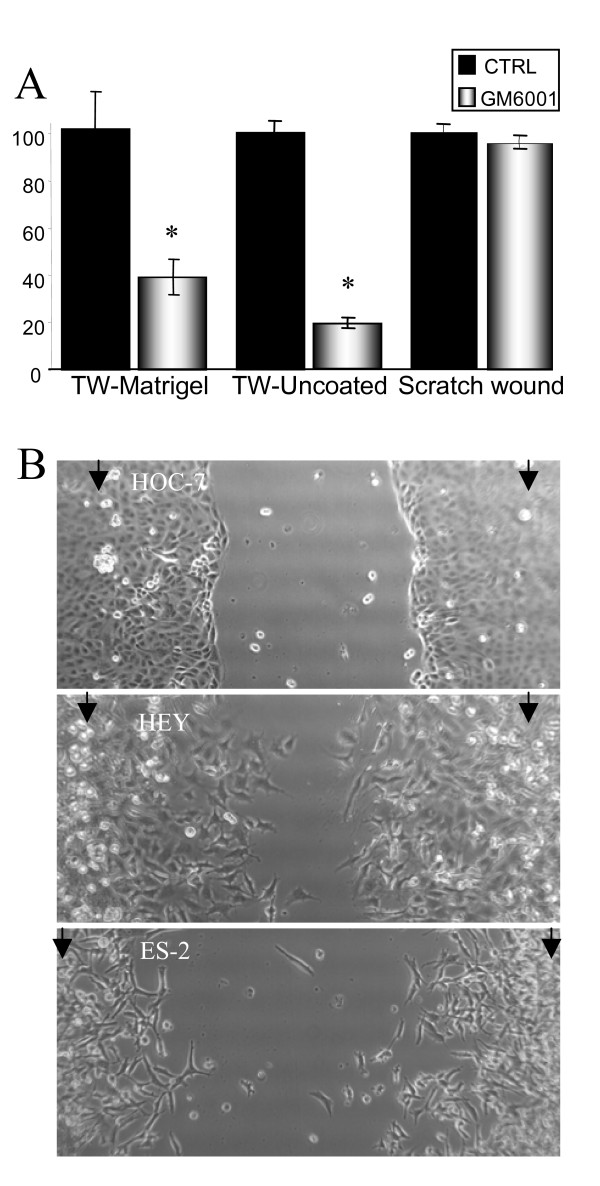
**Inhibition of HOC-7 transwell penetration by MMP inhibition is unrelated to the Matrigel coating.***A*. Effect of GM6001 on different modes of HOC-7 migration. *B*. Cell morphology and migration mode during scratch-wound healing assay at 10 hrs after wounding. Arrows indicate the borders of the scratch at time 0. HOC-7 cells have epithelial morphology and migrate as a sheet, in contrast to fibroblastic-like HEY and ES-2 cells which undergo single-cell migration. Bars represent the mean ± S.E. *Significantly different from control (p < 0.05; 2-tailed t-test).

In comparison to HEY and ES-2 cells, the ability of HOC-7 ovarian cancer cells to penetrate Matrigel-coated transwells appeared particularly sensitive to MMP inhibition, showing a 60% reduction in response to GM6001 (p = 0.01; Fig. 3A). However, the migration of these cells across uncoated transwell membranes was also markedly reduced (80% reduction) in the presence of GM6001 (p = 2.2E-5), indicating that the Matrigel coating was irrelevant to the effect of MMP inhibition on HOC-7 transmigration (Fig. 3A). In contrast, HEY and ES-2 cell migration across uncoated transwell membranes was not reduced by GM6001, nor was cell proliferation altered (data not shown).

HOC-7 cells exhibit an epithelial morphology, grow in tight clusters, and express E-cadherin, which is in contrast to the fibroblast-like morphology of the N-cadherin-expressing HEY and ES-2 cells[31]. The behaviour of these cells lines in a scratch-wound assay indicated that whereas HEY and ES2 cells migrate as single cells, HOC-7 cells migrate as a sheet (Fig. 3B). We therefore postulated that MMP inhibition might interfere with the cell-cell detachment that would be required for HOC-7 cell movement through the narrow (8 μm) transwell pores, as MMPs have been implicated in E-cadherin cleavage [32,33]. The migration of HOC-7 cells in a scratch wound healing assay, which allows motility to be assessed without a need for cell-cell detachment, was unaffected by MMP inhibition (p = 0.27; Fig 3A), providing support for this hypothesis.

### MMP requirement for penetration of 3D matrices is matrix specific

With emerging evidence that cell behaviour differs in a 3D environment that may more accurately reflect the *in vivo *situation, there is an increased use of 3D cell culture systems. We therefore assessed invasion of cells through 3D matrices. The culture of cancer cells as spheroids may recapitulate the tumour environment more accurately than that provided by monolayer culture [34,35].

**Figure 4:**
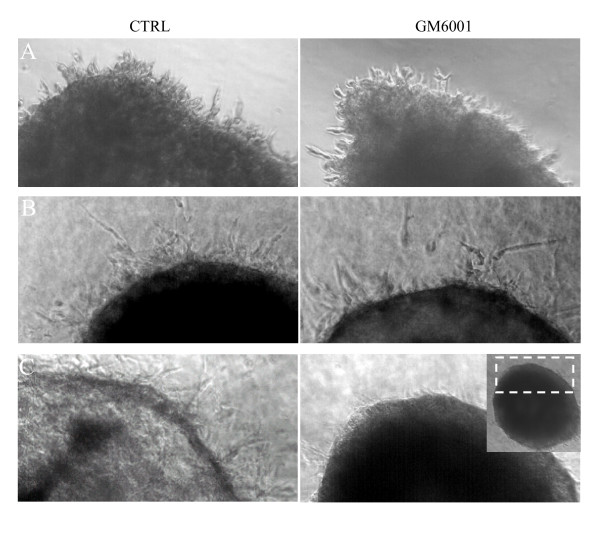
**MMP requirement for spheroid cell penetration into 3D matrices is matrix specific.** Ovarian cancer cell spheroids, generated by hanging drop culture, were embedded in polymerized matrices of *A*, Matrigel (undiluted, ~11 mg/ml) *B*, pepsin-extracted collagen I, (Vitrogen) and *C*, acid-extracted collagen I (2.1 mg/ml). Images reveal the spheroid edge, where cell penetration of matrices was apparent. An entire spheroid is shown in the lower inset. Cell penetration of Matrigel and pepsin-extracted collagen I (Vitrogen) occured despite MMP blockade with GM6001. In contrast, invasion of acid-extracted collagen 1 was prevented by MMP inhibition. Representative results are shown using OVCA429 spheroids at 60, 36, and 44 hrs for Matrigel, Vitrogen and acid-extracted collagen I, respectively.

3D spheroids generated through hanging drop culture [30] were embedded in Matrigel (~11 mg/ml) or pepsin-digested collagen I (Vitrogen; 2.1 mg/ml final concentration) and the effect of MMP inhibition on cell invasion was assessed. Cell migration into both Matrigel and Vitrogen matrices occurred despite MMP blockade. Because pepsin-digested collagen molecules lack the telopeptide domains required for cross-linking and matrix stability, we also performed these experiments using acid-extracted collagen I, which retains intact telopeptide domains [36,37]. In contrast to pepsin-digested collagen I, cell penetration of acid-extracted collagen I was abolished by MMP inhibition. Representative results are shown using OVCA429 (Fig. 4) with similar results obtained for HEY and ES-2 cells.

## Discussion

Establishing *in vitro *invasion assays that accurately reflect the circumstances *in vivo *is paramount to revealing mechanisms relevant to cancer metastasis. During peritoneal metastasis, cancer cells preferentially adhere to the submesothelial ECM rather than to the mesothelial cells [19,20]. The submesothelial connective tissue is periodically exposed at milky spots (lymphatic portals), which are prevalent on the surface of the omentum and the subdiaphragmatic peritoneum. In the early stages of peritoneal dissemination, cancer cells preferentially adhere to the milky spots [38] likely due to exposure of the underlying ECM at these sites [20,39]. Rather than effectively eliminating tumour cells, immune cells, including those at the milky spots, may promote tumour dissemination through their release of inflammatory mediators [40]. With cancer progression, the mesothelium becomes compromised [41] due to the action of inflammatory cytokines, including tumour necrosis factor α and interleukin 1β, which cause mesothelial cell retraction [20,42]. The widespread exposure of the underlying ECM causes a corresponding shift in the pattern of tumour cell attachment such that it is no longer limited to peritoneal surfaces with milky spots [20]. Therefore investigating cancer cell interaction with, and invasion of, the submesothelial ECM is vital. Unlike the archetypal basement membrane underlying true epithelia, consisting of a distinct collagen IV and laminin-rich sheet, the submesothelial ECM is not a typical basement membrane. [21]. Instead, collagen I and FN abut the mesothelial monolayers, co-localizing with thin deposits of collagen IV and laminin [21].

Based on its composition, Matrigel is believed to resemble basement membrane matrices, such as that of the endothelium, which cancer cells penetrate during haematological metastasis frequently used for *in vitro *invasion studies [9,10]. The attraction of the Matrigel invasion assay is that it provides a rapid, simple method to evaluate invasion within hours [5] as compared to the multiple days required for tumour cell invasion of intact basement membranes isolated from tissues. Despite the unique metastatic process and critical role of collagen I in peritoneal metastasis, the *in vitro *invasion of ovarian, gastric and colon cancer cells is also routinely assessed using Matrigel matrices. However, collagen I is comparable to Matrigel in terms of being commercially available and easy to prepare as a transwell coating or thick (3D) gel. Collectively, our experiments show that matrices reconstituted from Matrigel do not adequately reflect the barrier function of basement membranes, in either transwell or 3D invasion systems, whereas the barrier function of stromal matrices can be mimicked using polymerised collagen I. We show that ovarian cancer cells can migrate through Matrigel matrices, in the absence of MMP activity, in either thin 2D transwell or thick 3D spheroid invasion contexts. In contrast, ovarian cancer cell penetration through a collagen I barrier required MMP activity.

Matrigel differs from authentic basement membranes in terms of the relative abundance of and interactions between the components [43], and this may underly its inability to mimic the barrier function of intact basement membranes. Collagen IV is essential for basement membrane tensile strength and stability, forming a cross-linked network with which the laminin network interacts. Whereas the components of Matrigel are chemically and immunologically similar to the major components of basement membranes [44], the relative abundance of and interactions between the components differ. Most notably, Matrigel matrices are substantially less cross-linked than basement membranes [43]. As it is the cross-linked structure that imparts strength and integrity to intact basement membrane matrices, it follows that Matrigel matrices would have lower resistance to cell penetration and be unable to reflect the in vivo situation.

Collagen I is arguably the most important ECM component with which ovarian cancer cells interact during peritoneal dissemination. It is the preferred substrate for adhesion and migration of ovarian cancer cells and also stimulates their invasive behaviour. An abundant constituent of the peritoneal stromal matrix that is present directly beneath the mesothelium, collagen I is exposed to the peritoneal cavity at milky spots and as a result of mesothelial retraction. The importance of tumour cell interaction with collagen I in peritoneal metastasis is supported by the fundamental role of its receptors α2β1 integrin and α3β1 integrin [23,45,46]. Altogether, this provides strong rationale for the use of collagen I matrices in investigations pertaining to invasive behaviour by ovarian and other cancers that undergo peritoneal metastasis.

In 3D culture, MMP-mediated proteolysis was required for invasion of matrices formed from acid-extracted collagen I but not of matrices formed from pepsin-digested collagen I (Vitrogen/Purecol). Differences in the requirement for MMP activity for ovarian cancer cells to invade collagen I matrices formed from acid-extracted rat tail collagen versus pepsin-solubilized collagen may be attributed to differences in the structural integrity of the reconstituted collagen. Pepsin cleaves within the telopeptide regions of collagen I molecules (creating atelocollagen), whereas these domains remain intact when acid-extraction is used for solubilization. In addition to promoting collagen I assembly [47], intact telopeptide regions are critical to the strength and stability of fibrillar collagen I matrices through the provision of lysine residues required for intermolecular cross-link formation [36]. These covalent cross-links prevent collagen molecules from sliding past one another and are the basis of the tensile strength of collagen fibre systems. The collagen cross-links re-establish during reconstitution of the acid-extracted collagen I [37], but not pepsin-digested collagen I (atelocollagen) preparations. Consequently, it is conceivable that cancer cells should be able to migrate through the more compliant atelocollagen matrices without the requirement for its degradation. Interestingly, although MMP activity was not required for cell penetration of 3D gels of atelocollagen, it was required when the atelocollagen was dried onto transwells. This affixing of collagen to the transwell membrane may compensate for the lack of cross-linking, effectively preventing fibrils from sliding past one another as would occur in a 3D gel.

The barrier function of Matrigel was implied but not verified in the original description of the Matrigel chemoinvasion assay [10]. However, cell penetration of Matrigel does not always correlate with invasiveness. Studies have shown that normal fibroblasts are capable of passing through Matrigel, whereas many invasive epithelial cancer cell lines are not, and correlation between invasive potential and capacity for Matrigel penetration was found to be lacking [48-50]. Rather, cells of mesenchymal lineage had a superior capacity for Matrigel penetration compared to those of epithelial origin, regardless of whether they were malignant or had invasive potential *in vivo*. Moreover, extensive migration of the cells into Matrigel occurred in the absence of matrix degradation [48], which is consistent with our findings. Therefore, the use of Matrigel as an invasion matrix is inconsistent with the current dogma that MMPs are important for cancer cell invasion. Conversely, we have found that the *in vitro *collagen I invasion capacity of a panel of ovarian cancer cell lines reflected the *in vivo *capacity for peritoneal tumour formation reported by others [31].

The function of MMPs in matrix proteolysis is well established, yet this family of proteases also has critical roles in various additional physiological functions that include the cleavage of cell adhesion molecules and growth factors [51]. The results obtained for HOC-7 cells emphasize that MMP activity can influence Matrigel transmigration (in a cell line specific manner) for reasons unrelated to alleviation of a matrix barrier. The apparent marked GM6001-mediated inhibition of HOC-7 Matrigel "invasion" was paralleled by a comparable reduction in migration through uncoated transwells, yet HOC-7 scratch wound migration was unaffected by MMP inhibition. It is plausible that MMP inhibition blocks the cell-cell detachment required for these cells to migrate through the narrow (8 μm) transwell pores because in contrast to the fibroblast-like HEY and ES-2 cells, the HOC-7 cells have an epithelial morphology, grow in tight clusters, express E-cadherin [31], and migrate as a sheet. This interpretation is supported by evidence that E-cadherin cleavage is mediated by MMPs [1,32,33].

Matrigel contains numerous growth factors (TGFβ, EGF, FGF, PDGF, IGF [7]) that may be activated or released from the matrix by proteolytic cleavage, including by some MMPs. The chemotactic response to various agents differs between cells [52], and could contribute to observed differences in sensitivity to MMP inhibitors between cell lines (eg. HEY versus ES-2 in this study). This might explain why MMP inhibition has had, at best, only a modest influence on Matrigel penetration as compared to collagen I invasion.

The importance of considering auxiliary factors involved in the transmigration process that are unrelated to matrix penetration has been emphasized [52], yet most studies evaluate treatment effects for Matrigel penetration in the absence of migration controls. Thus, without careful examination, the requirement of MMPs for cell dissociation/migration in some cancer cells lines may have been misinterpreted as a requirement for proteolytic degradation of matrices. In exceptional studies where migration control experiments have been performed, treatments tended to affect Matrigel "invasion" and migration similarly (for example [53-55]). Thus, the impact on Matrigel penetration should be considered secondary to an altered motility unless alterations in matrix proteolysis are demonstrable.

Our results do not preclude the possibility that cells utilize alternative proteolytic systems for Matrigel penetration. For example, cathepsins have been implicated in transwell Matrigel penetration [54,56,57] by cancer cells, including ovarian [58]. However, marked reductions in cell motility have also been documented in response to cathepsin protease inhibition [54]. Therefore, it is unclear whether Matrigel degradation by cathepsin proteases is a requirement for cell penetration *per se*, or whether the observed reductions in Matrigel penetration merely reflect reduced cell motility. Although other proteolytic systems may influence invasion, it is widely accepted that MMP activity is a critical aspect of tumour penetration of basement membranes *in vivo*.

Recent studies suggest cancer cells can circumvent the requirement for matrix proteolysis and migrate through tissues by adopting an amoeboid form of movement [59-62]. It is notable that these studies were performed in 3D atelocollagen or Matrigel gels, not in acid-extracted collagen I. Whereas channels lined with collagen I degradation products were generated during cancer cell invasion of 3D acid-extracted collagen I matrices, such channels were not observed when pepsin-digested collagen was used [63]. Thus, the phenomenon of protease-independent tumour cell invasion may be limited to those matrices with low levels of cross-linking.

## Conclusion

Invasion involves two distinct cellular processes: matrix degradation and cell motility. In absence of a requirement for matrix degradation, an assay primarily evaluates motility. To qualify as a *bona fide *invasion assay it is critical to demonstrate a proteolytic dependence for matrix barrier removal. Thereafter, it is imperative to demonstrate that the inhibition of proteolytic activity does not affect cell migration on uncoated membranes. Under these conditions our studies indicate that the predominant behaviour evaluated in the Matrigel chemoinvasion assay is the capacity for cell migration on laminin/collagen IV and not MMP-mediated invasion that occurs through basement membranes and stromal matrices *in vivo*. In general, cell migration through Matrigel may indirectly reflect some aspects of invasive potential in that cell attachment to laminin and motility on this substratum are correlates of metastatic potential [64,65]. However, in contrast to carcinomas that undergo haematological metastasis and must penetrate the vascular endothelial basement membrane, ovarian cancer cell interaction with basement membrane components are not likely critical for peritoneal metastasis, as an archetypal basement membrane does not exist beneath the peritoneal mesothelium [21]. Therefore, although Matrigel provides a highly useful, accessible extracellular matrix for the investigation of numerous cell behaviours, its use as a surrogate basement membrane to measure invasion is questionable. Collagen I is the preferred substrate for ovarian cancer attachment migration and invasion, is abundant beneath the mesothelium, and can be reconstituted to form matrices that provide a barrier function. Taken together, collagen I rather than Matrigel should be used in studies investigating ovarian cancer invasive behaviours to better approximate critical interactions and events associated with peritoneal metastasis.

## Competing interests

The authors declare that they have no competing interests.

## Authors' contributions

KLS performed all studies and drafted the manuscript. TJB performed statistical analysis. TJB and MJR revised the manuscript. All authors were involved in the conception of the study and data interpretation. All authors have read and approved the final manuscript.

## Pre-publication history

The pre-publication history for this paper can be accessed here:


